# Effects of school-based physical activity on academic achievement in children and adolescents: a systematic review and meta-analysis

**DOI:** 10.3389/fpubh.2025.1651883

**Published:** 2025-09-16

**Authors:** Hongxue He, Yahui Yang, Jiaojiao Sun, Fang Wang, Wei Zhang, Fengshu Zhu

**Affiliations:** ^1^College of Physical Education, Yangzhou University, Yangzhou, Jiangsu, China; ^2^Wenhe Primary School of Yangzhou, Jiangsu, China

**Keywords:** physical activity, academic achievement, children, adolescents, meta-analysis

## Abstract

**Objective:**

This study explores how school-based physical activity affects the academic achievement of children and adolescents and examines whether factors like activity type or duration influence results.

**Method:**

We registered the review in PROSPERO (CRD42024623670). We searched PubMed, Web of Science, Cochrane Library and Embase databases for peer-reviewed English-language randomized or quasi-experimental studies published through 8 December 2024. Reference lists and gray literature were reviewed. The Cochrane Risk of Bias Tool assessed study quality, with findings analyzed through subgroup and sensitivity analysis. Review Manager 5.4 calculated the standardized mean difference (SMD) and 95% confidence interval (CI) using a random-effects model.

**Results:**

Seventeen studies met inclusion criteria. Meta-analysis indicated physical activity programs significantly improved academic achievement, especially in mathematics (SMD = 0.11, 95% CI: 0.04–0.18, *p* = 0.001; *I^2^* = 55%) and overall academic achievement (SMD = 0.22, 95% CI: 0.01–0.44, *p* = 0.040; *I^2^* = 74%). Subgroup analysis revealed moderate-intensity activity positively correlated with mathematics (SMD = 0.08, 95% CI: 0.01–0.15, *p* = 0.040; *I^2^* = 37%) and overall academic achievement (SMD = 0.36, 95% CI: 0.14–0.57, *p* = 0.001; *I^2^* = 70%) results. High-intensity activity showed a positive effect on mathematics (SMD = 0.41, 95% CI: 0.14–0.68, *p* = 0.003; *I^2^* = 6%). Short-duration (<24 weeks) interventions positively impacted reading comprehension (SMD = 0.24, 95% CI: 0.03–0.46, *p* = 0.030; *I^2^* = 69%), while longer interventions (≥24 weeks) improved mathematics (SMD = 0.13, 95% CI: 0.03–0.23, *p* = 0.010; *I^2^* = 66%) and overall academic achievement (SMD = 0.47, 95% CI: 0.25–0.68, *p* < 0.001; *I^2^* = 14%). Despite these significant findings, substantial heterogeneity was observed in several analyses, indicating that the results should be interpreted with caution.

**Conclusion:**

School-based physical activity positively impacts academic achievement, particularly in mathematics and overall performance. Longer, moderate-intensity interventions are most effective, offering insights for future educational program development.

**Systematic review registration:**

https://www.crd.york.ac.uk/prospero/display_record.php?ID=CRD42024623670, CRD42024623670.

## 1 Introduction

Academic achievement is commonly defined as the extent to which teachers, students, or educational institutions attain educational objectives, typically measured through examinations or continuous assessments (1). Currently, significant fluctuations in student performance are observed globally (2). Evidence suggests that students with different levels of academic achievement exhibit varying psychological health outcomes. Those with lower academic achievement are at greater risk of internalizing problems during adolescence, including emotional, psychological, and behavioral issues (3). In contrast, students with higher academic achievement may experience direct mental health benefits by enhancing psychosocial resources such as self-esteem (4). Academic achievement not only affects students’ educational advancement but may also have long-term implications for career development, health outcomes, and socioeconomic status (5).

Physical activity is defined as any bodily movement produced by skeletal muscles that requires energy expenditure. Currently, most children and adolescents do not engage in sufficient levels of physical activity. According to statistics from the World Health Organization, 81% of adolescents fail to meet the WHO’s recommendation of an average of 60 min per day of moderate-to-vigorous physical activity (6). In recent years, increasing attention has been paid to the relationship between physical activity and academic achievement. Studies have shown that regular physical activity can improve academic performance by enhancing students’ attention, memory, and motivation to learn (7). In contrast, irregular physical activity tends to have a minimal and inconsistent impact on academic achievement (8). However, students who participate in moderate-intensity physical activity over a prolonged period show the most significant improvements in academic outcomes (9).

However, schools represent one of the most critical settings for promoting physical activity among children and adolescents (10). School-based physical activity refers to purposeful, planned, and organized physical exercises and movement forms conducted within the school setting, with enrolled students as the primary participants. In this study, school-based physical activity primarily includes physical education classes, structured recess-time physical activities, organized calisthenics during breaks, and physically active learning integrated into academic subjects (11). Extracurricular sports programs or spontaneous physical activities occurring after school hours are excluded. This distinction is critical for accurately assessing the effects of the interventions. Numerous studies have indicated that academic achievement results from the interplay of multiple factors across school, family, society, and the individual, with individual-level factors playing a particularly crucial role. School-based physical activity is closely associated with students’ mental health, self-efficacy, and learning motivation—key components of psychological capital (12, 13). Physical activity contributes significantly to brain development during childhood and adolescence. It enhances synaptic connectivity, cerebral blood flow, and BDNF expression, while promoting neurogenesis in regions linked to learning and executive function. These changes support academic performance by improving memory, attention, flexibility, and processing speed. Different types of physical activity, including aerobic exercise, resistance training, and combined programs, engage distinct neural mechanisms. Aerobic exercise supports hippocampal and vascular health, while resistance training affects hormonal balance. Combined approaches offer broader cognitive benefits (14, 15). In school settings, structured physical activity also fosters higher-order cognitive skills. Programs designed to engage students physically have been shown to enhance creativity and problem-solving, promoting adaptability and innovation in learning contexts (16, 17). However, findings on the effects of school-based physical activity on academic achievement remain inconsistent. Sun (18) reported that students with higher academic performance tended to participate less frequently in school-based physical activities and engaged in activities of lower intensity (18). These discrepancies in research outcomes may stem from the diversity in physical activity formats and variations in study designs related to physical activity variables such as intensity, duration, frequency, and intervention period. Additionally, differences in the criteria and methods used to assess academic achievement may also contribute to the variability in results.

Currently, there is a paucity of intervention studies focusing on the effects of school-based physical activity on academic achievement. Most existing research has focused on evaluating the overall effectiveness of school-based physical activity, while offering limited analysis of how specific intervention characteristics—such as intensity, duration, or frequency—may influence academic achievement. In addition, findings across studies have been inconsistent. Some evidence suggests that participation in school-based physical activity may increase academic stress and physical fatigue, potentially leading to a decline in academic achievement (19). Therefore, this study employs a systematic review and meta-analysis to comprehensively assess the impact of school-based physical activity interventions on academic achievement among children and adolescents. Specifically, this study aims to evaluate the impact of school-based physical activity on academic outcomes among children and adolescents, and to examine how differences in intervention characteristics (e.g., intensity, duration) may be associated with academic achievement. This work seeks to address limitations in previous meta-analyses and provide a theoretical foundation for conducting effective experimental research and implementing evidence-based physical activity programs in school settings.

## 2 Methods

### 2.1 Search strategy

The PROSPERO registration number for the study protocol is CRD42024623670. A search was performed across four databases: Web of Science, PubMed, Cochrane Library and Embase. The search encompassed literature published in English from the foundation of each database to December 8, 2024, following peer review. The search parameters encompassed: (a) Adolescence OR Adolescents OR Female Adolescent OR Female Adolescents OR Male Adolescent OR Male Adolescents OR Youth OR Youths OR Teens OR Teen OR Teenagers OR Teenager OR Child OR Children; (b) Exercise OR Acute Exercise OR Aerobic Exercise OR Exercise Training OR Isometric Exercise OR Physical Activity; (c) Academic Success OR Academic Achievement OR Academic Achievements OR Academic Successes. Based on database features, Boolean logic was used to search. Additionally, chosen study reference lists and gray literature were rigorously evaluated to find papers that met inclusion criteria. To further reduce potential bias and supplement articles that may have been missed by database indexing, we also manually screened the reference lists of all included studies and relevant systematic reviews.

### 2.2 Inclusion and exclusion criteria

The inclusion criteria for this study, based on the PICOS (Population, Intervention, Comparison, Outcome, Study Design) framework for systematic reviews, are as follows:

1. The population comprises children and adolescents aged 6–18 years (Population).
2. The school-based physical activity interventions included in this review covered PE classes, active classroom breaks, and physically integrated academic lessons. Although these formats vary in structure, frequency, and intensity, they share key characteristics: they are implemented during regular school hours, supervised by school staff, and designed to engage students physically within the educational context. The inclusion of diverse modalities reflects real-world practices but may also introduce heterogeneity in underlying mechanisms, such as differences in cognitive engagement, motor demands, or instructional goals (Intervention).
3. The comparison group consists of regular physical education classes in which no specific training content is provided (Comparison).
4. The outcome measure is academic achievement. Academic achievement is assessed through non-standardized tests, including scores in mathematics, reading comprehension, spelling, language expression, and overall academic achievement. These instruments differ in scope and evaluative focus: non-standardized tests typically assess norm-referenced performance, while teacher grades may reflect a combination of academic progress, classroom behavior, and effort. To ensure comparability across studies, outcomes were synthesized using standardized mean differences (SMD). The diversity of measurement approaches is recognized as a potential source of heterogeneity; therefore, subgroup analyses were conducted, where feasible, to examine effects by outcome type. Spelling is a language expression skill that requires integration of phonology, orthography, and morphology. This integrative capability is a significant indicator of linguistic proficiency, and the enhancement of spelling skills is intricately linked to expressive language abilities (20). Therefore, spelling is categorized as part of language expression in the data extraction process. Academic achievement ultimately includes four components: mathematics, reading comprehension, language expression, and overall academic achievement. The overall academic achievement is calculated as the average score across subjects or evaluated by teachers according to specific national curriculum assessment standards for each subject (Outcome).
5. The study design is a randomized controlled trial (Study Design).

Exclusion criteria: (1) Unpublished literature; (2) Incomplete or non-mergeable outcome data; (3) Conference abstracts, theses, or duplicate publications; (4) Studies involving populations with developmental abnormalities (e.g., Down syndrome).

### 2.3 Study selection

In accordance with PRISMA guidelines, two reviewers independently conducted the literature screening and data extraction. All identified studies were imported into Zotero for deduplication. The reviewers then applied the predetermined inclusion and exclusion criteria to titles, abstracts, and subsequently full texts. In cases of disagreement regarding study inclusion, the two reviewers first discussed the conflict in detail to reach a shared interpretation of the criteria. If consensus was not achieved, a third senior reviewer independently evaluated the disputed study. Final inclusion decisions were made through a consensus meeting among all three reviewers. For studies with missing full texts or insufficient data, we contacted the corresponding authors via email. If no response was received within 2 weeks, the study was excluded. All extracted data were entered into Excel and cross-verified by two researchers to ensure accuracy and consistency.

In this study, school-based physical activity interventions were separated from outdoor physical activities to more precisely assess the independent effects of school-based interventions. Although this approach provides a clearer evaluation of intervention effects, we acknowledge that future research could explore the combined impact of school interventions and outdoor physical activities. Furthermore, studies could adopt standardized metrics to quantitatively examine the relationship between physical activity and academic achievement. This distinction allows for an isolated examination of school interventions but may limit the overall understanding of the comprehensive effect of physical activity on academic outcomes.

### 2.4 Data extraction

Two researchers independently extracted data on sample descriptions (author, year, country, and participant characteristics), outcome measurements, and intervention parameters (e.g., frequency, duration, and type of physical activity). In addition to baseline values and baseline changes, the mean (M) and standard deviation (SD) of baseline and endpoint outcome measures were extracted. If precise data for merging or conversion were not available, the final mean and standard deviation were estimated using the Cochrane Handbook version 5.1.0 techniques after a discussion (21).

### 2.5 Risk of bias assessment

The risk of bias was independently assessed by two reviewers using the Cochrane Collaboration Risk of Bias Tool, covering seven domains: random sequence generation, allocation concealment, blinding of participants and personnel, blinding of outcome assessment, incomplete outcome data, selective reporting, and other sources of bias. Any disagreements were resolved through discussion or by consultation with a third reviewer.

### 2.6 Data analysis

All data were subjected to statistical analysis utilizing Review Manager 5.4 software. Given the variability in study design, participant characteristics, intervention types, and outcome measures, a random-effects model (DerSimonian–Laird method) was applied to all meta-analyses to account for between-study heterogeneity and provide more conservative effect estimates (22). The *I^2^* statistic and *p*-values were used to assess statistical heterogeneity in the meta-analysis. *I^2^* values of 25, 50, and 75% indicate heterogeneity levels of minor, moderate, and large degrees (23). All outcome indicators included in the analysis were continuous variables, and results were reported using standardized mean differences (SMD) and 95% confidence intervals (CI). When *I^2^* > 50%, substantial heterogeneity was considered present, and a random-effects model was applied. Sensitivity analyses or subgroup analyses were conducted to explore potential sources of heterogeneity and enhance result consistency. Results were considered statistically significant when *p* < 0.05.

## 3 Results

### 3.1 Study characteristics and risk of bias

**Figure 1:**
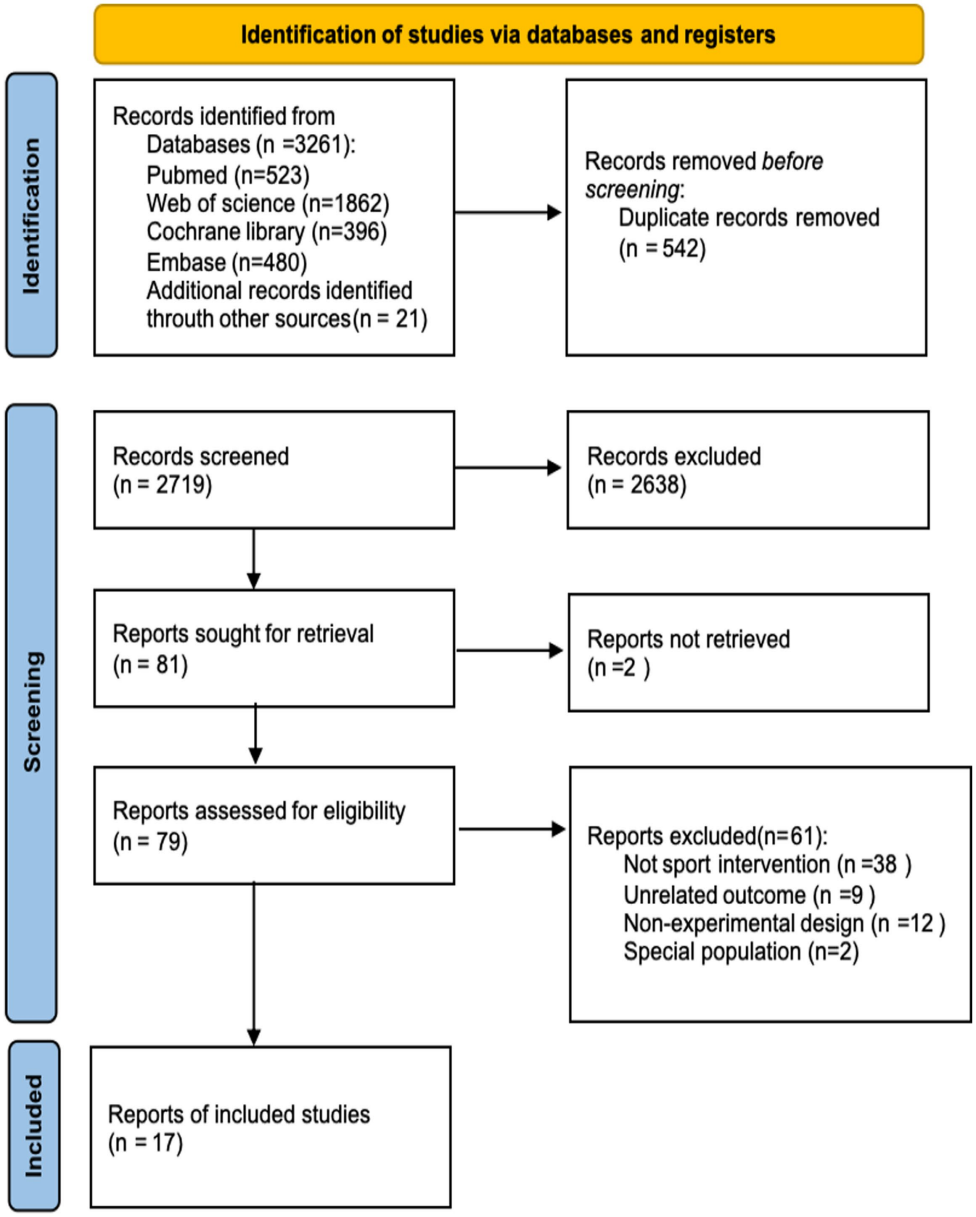
Flowchart of the selection process.

**Figure 2:**
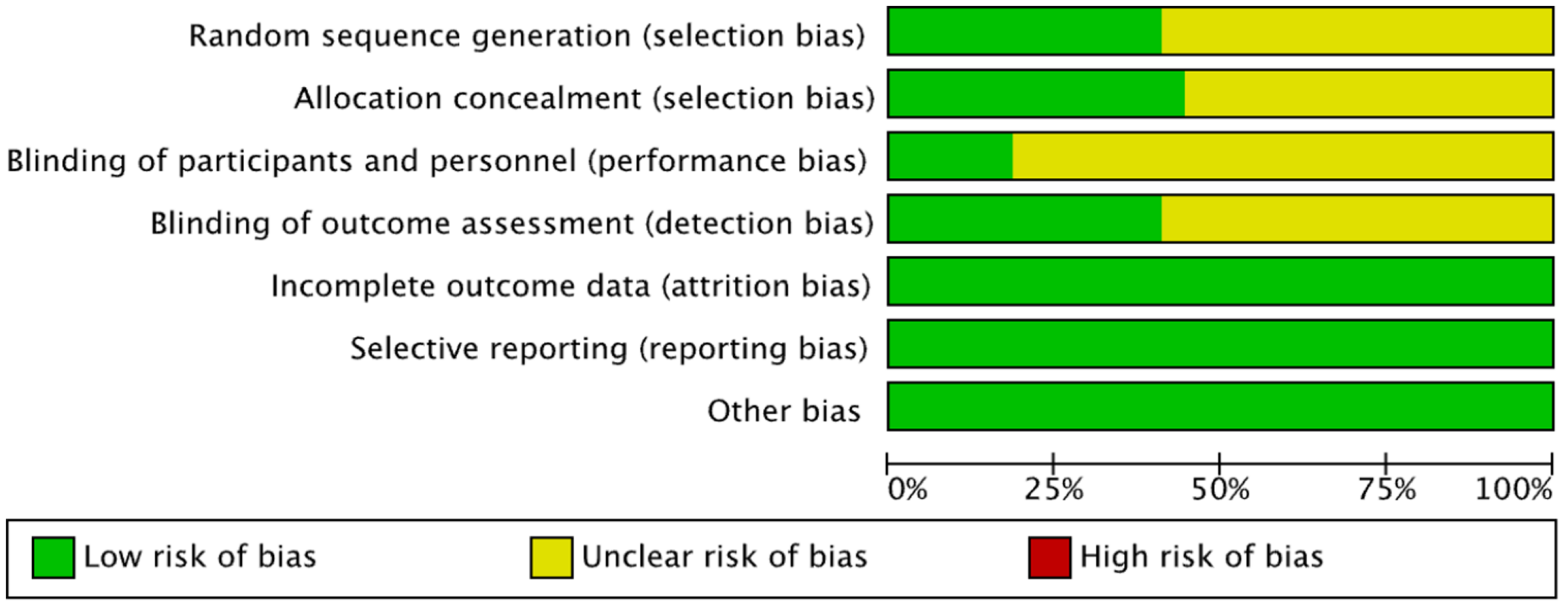
Risk of bias graph.

After screening, this review included 17 studies (24–40). Figure 1 illustrates the comprehensive screening procedure, and the included randomized controlled trials (RCTs) were published between 2007 and 2024. All investigations were RCTs. Twelve studies documented mathematics achievement, five documented reading comprehension achievement, six documented language expression achievement, and five documented overall academic achievement. Intervention durations ranged from 4 weeks to 3 years. A complete screening technique is shown in Table 1. All studies rated “moderate quality,” with detailed assessments in Figure 2.

**Table 1:** Characteristics of included studies.

Study, design, country	Participants, age, sample size (intervention group/control group)	Intervention group	Control group	Intervention frequency, duration, & intensity	Outcomes
Ahamed et al. (24) RCT Vancouver	Grade 4–5 elementary school students 10.2 (0.6) 214/74	Two regular PE lessons per week, an additional 15 min of daily classroom-based physical activity (total 150 min/week)	Two regular PE lessons per week (40 min per session, total 80 min/week), no additional intervention	64 weeks, Moderate intensity	d
Ardoy et al. (25) IG1 RCT Spain	High school students 13 (0.1) 29/29	Four PE lessons per week (same content as the control group)	Regular PE lessons (twice per week, 55 min per session)	16 weeks, Moderate intensity	a, c
Ardoy et al. (25) IG2 RCT Spain	High school students 13 (0.1) 29/29	Four high-intensity PE lessons per week (HR > 120 bpm)	Regular PE lessons	16 weeks, High intensity	a, c
Beck et al. (26) IG1 RCT Denmark	Grade 1 students 7.5 (0.26) 53/57	Fine motor activities integrated with math (e.g., LEGO manipulations)	Traditional math teaching without any physical activity	6 weeks, Three times per week, 60 min per session, Moderate intensity	a
Beck et al. (26) IG2 RCT Denmark	Grade 1 students 7.5 (0.26) 55/57	Gross motor activities integrated with math (e.g., jumping, balancing, crawling)	Traditional math teaching without any physical activity	6 weeks, Three times per week, 60 min per session, Moderate intensity	a
De Bruijn et al. (27) IG1 RCT Netherlands	Grade 3–4 elementary school students 9.17 (0.66) 214/417	Moderate-intensity physical activities (e.g., running, relay races, sit-ups)	Two PE lessons per week	14 weeks, Four times per week, 30 min per session, Moderate intensity	a, b, c
De Bruijn et al. (27) IG2 RCT Netherlands	Grade 3–4 elementary school students 9.17 (0.66) 237/417	Cognitive-challenging activities combined with motor skills (e.g., dodgeball, climbing, balance beam, complex rules, coordination exercises)	Regular PE lessons	14 weeks, Four times per week, 30 min per session, Moderate intensity	a, b, c
Donnelly et al. (28) RCT USA	Grade 2–3 elementary school students 8.1 (0.6) 316/268	Classroom-integrated physical activities covering math, language arts, geography, spelling	Regular PE lessons	144 weeks, Three times per week, 55 min per session, Moderate intensity	a, b, c
Egger et al. (29) IG1 RCT Australia	Children aged 7–9 7.87 (0.39) 47/29	High cognitive engagement, high physical movement activities	No intervention	20 weeks, Twice daily, 10 min per session, High intensity	a, b, c
Egger et al. (29) IG2 RCT Australia	Children aged 7–9 7.87 (0.39) 49/29	High physical activity, low cognitive engagement	No intervention	20 weeks, Twice daily, 10 min per session, High intensity	a, b, c
Egger et al. (29) IG3 RCT Australia	Children aged 7–9 7.87 (0.39) 46/29	High cognitive engagement, low physical movement	No intervention	20 weeks, Twice daily, 10 min per session, High intensity	a, b, c
Elish et al. (30) RCT USA	Grade 4 students 7.87 (0.39) 29/29	Seven times per week, 30 min physical activity	Regular physical activity	52 weeks, Moderate intensity	a, b, c
Gall et al. (31) RCT Switzerland	Grade 4 students 8–13 years old 265/398	Two PE lessons and one dynamic music lesson per week	No physical activity intervention	20 weeks, twice weekly, 45 min per session, Moderate intensity	d
Garst et al. (32) RCT South Carolina	Grade 6–8 students 12.73 (0.94) 70/71	High-intensity fitness training program	Traditional PE lessons	36 weeks, Three times per week, 60 min per session, High intensity	d
Lima et al. (33) IG1 RCT Austria	Grade 10 students 14.99 (1.04) 242/188	Four PE lessons per week, 3 h total	Regular PE lessons, two per week, 1.5 h total	24 weeks, Moderate intensity	a
Lima et al. (33) IG2 RCT Austria	Grade 10 students 14.99 (1.04) 198/188	Five seminar sessions per week, 4 h per session	Regular PE lessons, two per week, 1.5 h total	24 weeks, Low intensity	a
Lima et al. (33) IG3 RCT Austria	Grade 10 students 14.99 (1.04) 132/188	Double PE lesson seminars	Regular PE lessons, two per week, 1.5 h total	24 weeks, Moderate intensity	a
Mavilidi et al. (34) IG1 RCT Australia	Elementary students 9.11 (0.62) 29/29	Break-time activity, watching videos and mimicking actions	Traditional math course	4 weeks, Three times per week, 5 min per session, Moderate intensity	a
Mavilidi et al. (34) IG2 RCT Australia	Elementary students 9.11 (0.62) 29/29	Activity integrated with math lessons	Traditional math course	4 weeks, Three times per week, 5 min per session, Moderate intensity	a
Mavilidi and Vazou (35) IG1 RCT Lowa	Elementary students 9–11 years old 221/205	Physical activity integrated with math lessons	Traditional math courses, no physical activity	8 weeks, Three times per week, 10–12 min per session, Moderate intensity	a
Mavilidi and Vazou (35) IG2 RCT Iowa	Elementary students 9–11 years old 134/205	Physical activity unrelated to academic content	Traditional math courses, no physical activity	8 weeks, Three times per week, 10–12 min per session, Moderate intensity	a
Melero et al. (36) RCT Spain	14.63 (1.38) 113/37	TPSR-based and gamification strategies	Regular PE lessons	36 weeks, Twice weekly, 55 min per session, Low intensity	a, c, d
Mullender-Wijnsma et al. (37) RCT Lawrence, KS	8.1540/499	Physical activity integrated with math and language lessons	Traditional classroom teaching (sedentary learning method)	96 weeks, Three times per week, 20–30 min per session, Low intensity	a, b, c
Pinto-Escalona et al. (38) RCT Europe	Grade 2 students 7.4 (0.45) 29/29	Two hours of school-based karate intervention per week	Regular PE lessons	48 weeks, Moderate intensity	d
Ramos et al. (39) RCT Portugal	Grade 2 students 7.09 (0.29) 29/37	Physical activity integrated with math lessons	Traditional math lessons, no physical activity	12 weeks, Once per week, 45 min per session, Moderate intensity	a
Solberg et al. (40) IG1 RCT Norway	30 schools’ students 13.97 (0.3) 491/483	Academic Physical Education Class 30 min, Regular Physical Education Class 60 min, Physical Activity Class 30 min	Regular PE lessons, 120–180 min per week, no additional physical activity	36 weeks, Three times per week, Moderate intensity	a, b, d
Solberg et al. (40) IG2 RCT Norway	30 schools’ students 13.97 (0.3) 332/483	Happy Activity Class 60 min, Do not worry be happy (DWBH) 60 min	Regular PE lessons	36 weeks, Twice weekly, Low intensity	a, b, d

^a^Mathematics achievement, ^b^Reading comprehension achievement, ^c^Language expression achievement, ^d^Overall academic achievement.

### 3.2 The effect of interventions on mathematics achievement

**Figure 3:**
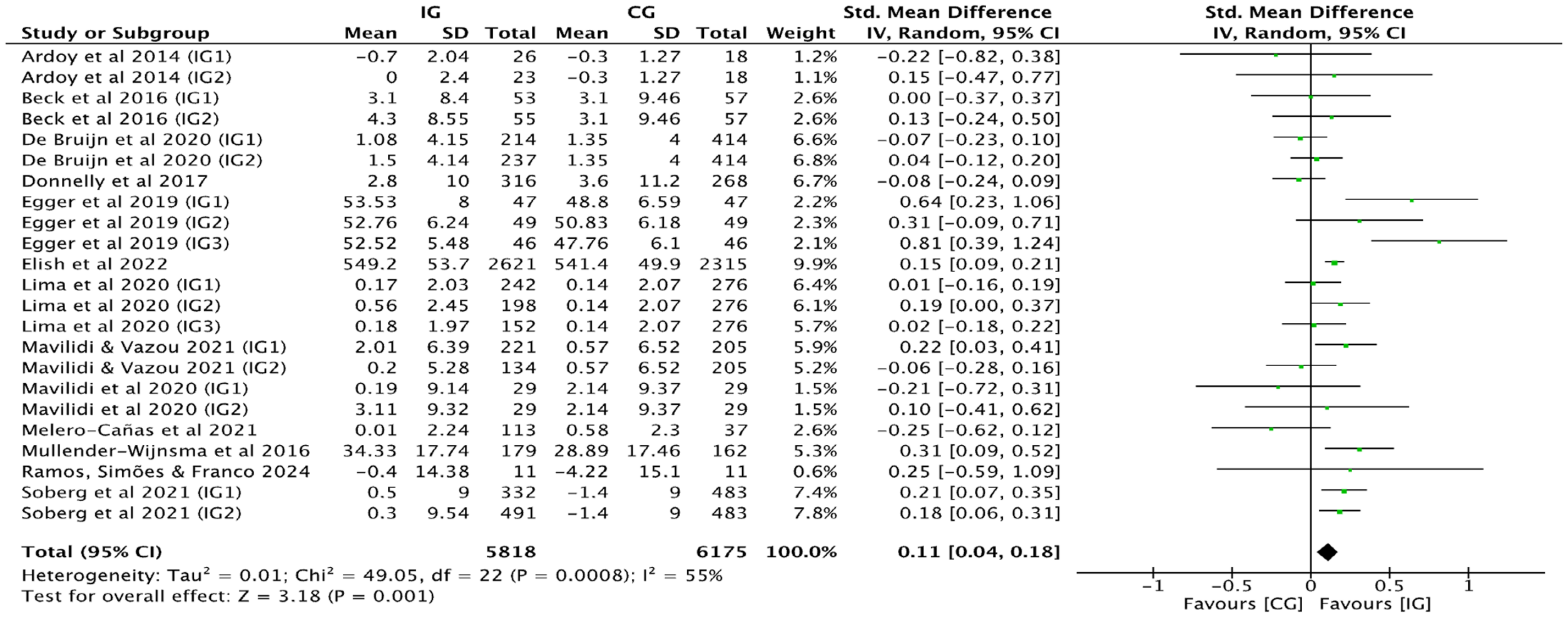
Forest plot of mathematics achievement.

Figure 3 shows that the combined effect size of 13 studies (25–30, 33–37, 39, 40) was SMD = 0.11, 95% CI: 0.04–0.18, *p* = 0.001, indicating a significant positive impact of physical activity on mathematics achievement. The pooled effect size for mathematics achievement demonstrated moderate heterogeneity (*I^2^* = 55%, *p* = 0.001), suggesting variability across studies. This may reflect differences in intervention types, durations, or delivery formats. To further explore potential sources of heterogeneity, subgroup analyses were conducted based on intervention intensity and duration, as reported in subsequent sections.

Subgroup analysis by intervention duration was performed (Table 2). When intervention duration <24 weeks, school-based physical activity was positively associated with mathematics achievement (SMD = 0.11, 95% CI: 0.01–0.21, *p* = 0.030; *I^2^* = 49%), suggesting that even relatively short-term interventions may yield academic benefits. However, a favorable association was found between physical activity and mathematics achievement after an intervention duration ≥24 weeks (SMD = 0.13, 95% CI: 0.03–0.23, *p* = 0.010; *I^2^* = 66%). An analysis of intervention intensity was also conducted (Table 2). Low-intensity physical activity did not significantly impact mathematics achievement (SMD = 0.13, 95% CI: −0.02 to 0.29, *p* = 0.080; *I^2^* = 71%). Mathematics achievement was positively correlated with moderate intensity physical activity (SMD = 0.08, 95% CI: 0.01–0.15, *p* = 0.040; *I^2^* = 37%) and strongly correlated with high-intensity physical activity (SMD = 0.41, 95% CI: 0.14–0.68, *p* = 0.003; *I^2^* = 6%).

**Table 2:** Subgroup analysis of mathematics, reading comprehension, language expression, and overall academic achievement.

Subgroup	Type	Number of studies	SMD (effect)	95% CI	*p*	*I*^2^ (%)
Mathematics achievement						
Intervention intensity	<24 weeks	17	0.11	[0.01, 0.21]	0.030	49
	≥24 weeks	6	0.13	[0.03, 0.23]	0.010	66
Intervention duration	Low intensity	8	0.13	[−0.02, 0.29]	0.080	71
	Moderate intensity	12	0.08	[0.01, 0.25]	0.040	37
	High intensity	3	0.41	[0.14, 0.68]	0.003	6
Reading comprehension achievement						
Intervention intensity	<24 weeks	5	0.24	[0.03, 0.46]	0.030	69
	≥24 weeks	5	−0.01	[−0.11, 0.10]	0.870	74
Intervention duration	Low intensity	3	0.12	[−0.02, 0.25]	0.100	22
	Moderate intensity	5	−0.01	[−0.12, 0.09]	0.790	73
	High intensity	2	0.50	[0.22, 0.79]	<0.001	0
Language expression achievement						
Intervention intensity	<24 weeks	7	−0.07	[−0.22, 0.09]	0.400	42
	≥24 weeks	4	−0.04	[−0.26, 0.18]	0.710	86
Intervention duration	Low intensity	3	0.05	[−0.29, 0.39]	0.770	69
	Moderate intensity	4	−0.08	[−0.24,0.09]	0.370	75
	High intensity	4	−0.15	[−0.43,0.14]	0.310	60
Overall academic achievement						
Intervention intensity	<24 weeks	4	0.13	[−0.11,0.37]	0.300	70
	≥24 weeks	3	0.47	[0.25, 0.68]	<0.001	14
Intervention duration	Moderate intensity	4	0.36	[0.14, 0.57]	0.001	70
	High intensity	2	0.03	[−0.56, 0.60]	0.920	63

**Figure 4:**
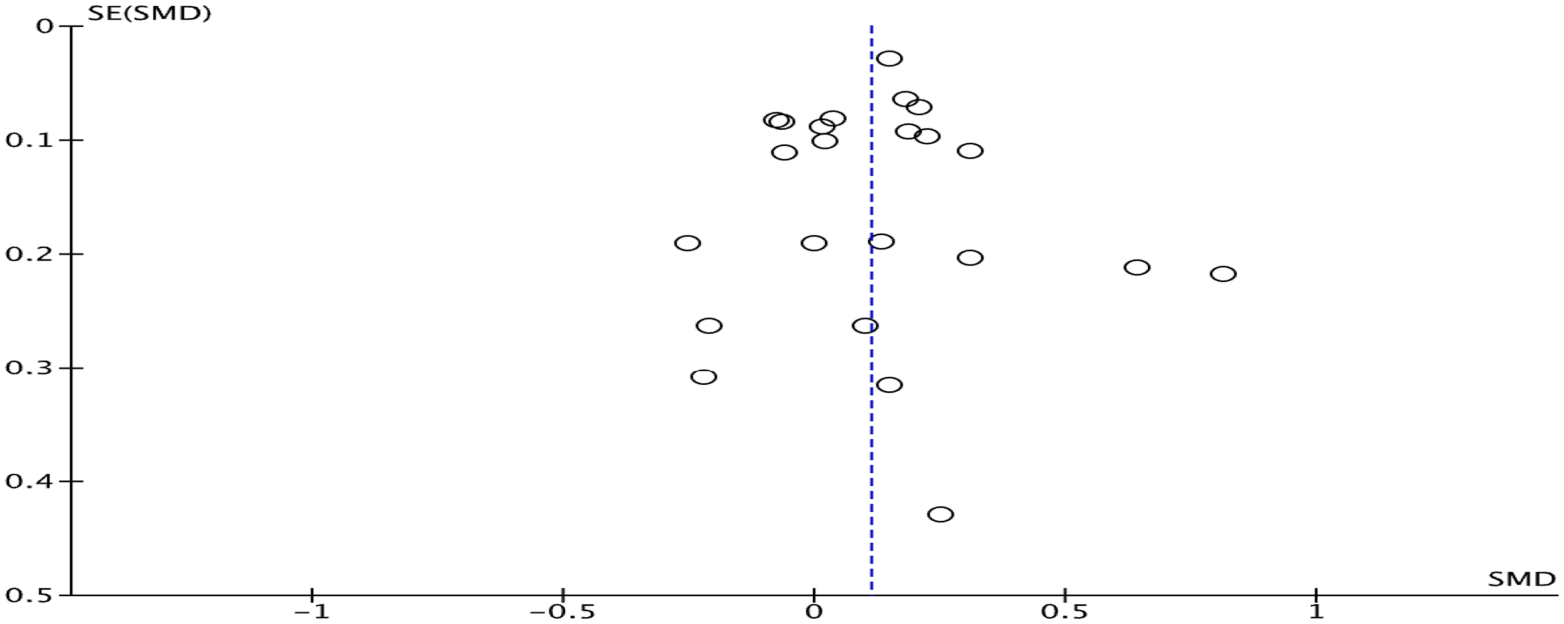
Funnel plot of mathematics achievement.

When any study was removed, and the effect size was recalculated, it was found that excluding Egger et al. (29) resulted in a decrease in heterogeneity (*I^2^* = 46%, *p* = 0.010). However, this study did not exhibit any characteristics. Heterogeneity in the other studies did not show significant changes before and after removal, as shown in Table 3, and had no substantial effect on the results. This suggests that the results of studies on the impact of physical activity interventions on mathematics achievement have low sensitivity, indicating that the meta-analysis results are stable. Funnel plot analysis was conducted to assess publication bias (Figure 4). The funnel plot for mathematics achievement appeared roughly symmetrical, with no apparent publication bias.

**Table 3:** Sensitivity analysis of mathematics, reading comprehension, language expression, and overall academic achievement.

Removed study	SMD (95%CI)	*p* (Total effect)	*I* ^2^	*p*
Mathematics achievement				
Ardoy et al. (25) IG1	0.12 (0.05, 0.18)	0.001	56%	<0.001
Ardoy et al. (25) IG2	0.11 (0.04, 0.18)	0.002	57%	<0.001
Beck et al. (26) IG1	0.11 (0.04, 0.19)	0.001	57%	<0.001
Beck et al. (26) IG2	0.11 (0.04, 0.18)	0.002	57%	<0.001
De Bruijn et al. (27) IG1	0.12 (0.05, 0.19)	<0.001	52%	0.003
De Bruijn et al. (27) IG2	0.12 (0.04, 0.19)	0.020	56%	<0.001
Donnelly et al. (28)	0.12 (0.06, 0.19)	<0.001	51%	0.003
Egger et al. (29) IG1	0.10 (0.03, 0.17)	0.003	51%	0.003
Egger et al. (29) IG2	0.11 (0.04, 0.18)	0.003	56%	<0.001
Egger et al. (29) IG3	0.10 (0.04, 0.16)	0.002	46%	0.010
Elish et al. (30)	0.11 (0.03, 0.19)	0.008	56%	<0.001
Lima et al. (33) IG1	0.12 (0.05, 0.19)	0.001	56%	<0.001
Lima et al. (33) IG2	0.11 (0.03, 0.18)	0.004	57%	<0.001
Lima et al. (33) IG3	0.12 (0.05, 0.19)	0.001	56%	<0.001
Mavilidi et al. (34) IG1	0.12 (0.05, 0.19)	<0.001	56%	<0.001
Mavilidi et al. (34) IG2	0.11 (0.04, 0.18)	0.002	57%	<0.001
Mavilidi and Vazou (35) IG1	0.11 (0.03, 0.18)	0.004	56%	<0.001
Mavilidi and Vazou (35) IG2	0.12 (0.05, 0.19)	<0.001	55%	0.001
Melero et al. (36)	0.12 (0.05, 0.19)	<0.001	53%	0.002
Mullender-Wijnsma et al. (37)	0.10 (0.03, 0.17)	0.005	54%	0.001
Ramos et al. (39)	0.11 (0.04, 0.18)	0.002	57%	<0.001
Solberg et al. (40) IG1	0.11 (0.03, 0.18)	0.005	56%	<0.001
Solberg et al. (40) IG2	0.10 (0.03, 0.18)	0.005	56%	<0.001
Reading comprehension achievement				
De Bruijn et al. (27) IG1	0.09 (−0.02, 0.21)	0.120	77%	<0.001
De Bruijn et al. (27) IG2	0.06 (−0.05, 0.17)	0.280	74%	<0.001
Donnelly et al. (28)	0.11 (0.00, 0.21)	0.050	71%	<0.001
Egger et al. (29) IG1	0.05 (−0.05, 0.15)	0.320	74%	<0.001
Egger et al. (29) IG2	0.04 (−0.05, 0.14)	0.380	71%	<0.001
Egger et al. (29) IG3	0.06 (−0.05, 0.16)	0.290	74%	<0.001
Mullender-Wijnsma et al. (37)	0.08 (−0.03, 0.20)	0.160	77%	<0.001
Elish et al. (30)	0.10 (−0.02, 0.23)	0.100	71%	<0.001
Solberg et al. (40) IG1	0.08 (−0.04, 0.19)	0.210	76%	<0.001
Solberg et al. (40) IG2	0.07 (−0.05, 0.18)	0.240	75%	<0.001
Language expression achievement				
Ardoy et al. (25) IG1	−0.07 (−0.19, 0.05)	0.260	70%	<0.001
Ardoy et al. (25) IG2	−0.07 (−0.19, 0.05)	0.270	70%	<0.001
De Bruijn et al. (27) IG1	−0.06 (−0.20, 0.08)	0.370	71%	<0.001
De Bruijn et al. (27) IG2	−0.06 (−0.20, 0.08)	0.420	71%	<0.001
Donnelly et al. (28)	−0.01 (−0.13, 0.10)	0.810	57%	0.010
Egger et al. (29) IG1	−0.04 (−0.17, 0.08)	0.500	70%	<0.001
Egger et al. (29) IG2	−0.03 (−0.14, 0.09)	0.640	65%	0.002
Egger et al. (29) IG3	−0.05 (−0.18, 0.07)	0.420	71%	<0.001
Elish et al. (30)	−0.06 (−0.22, 0.11)	0.480	71%	<0.001
Melero et al. (36)	−0.05 (−0.18, 0.08)	0.460	71%	<0.001
Mullender-Wijnsma et al. (37)	−0.10 (−0.21, 0.01)	0.070	55%	0.020
Overall academic achievement				
Ahamed et al. (24)	0.18 (−0.08, 0.43)	0.170	78%	<0.001
Ardoy et al. (25) IG1	0.24 (0.01, 0.46)	0.040	78%	<0.001
Ardoy et al. (25) IG2	0.21 (−0.02, 0.44)	0.080	79%	<0.001
Gall et al. (31)	0.15 (−0.05, 0.53)	0.140	52%	0.060
Garst et al. (32)	0.31 (0.12, 0.50)	0.002	63%	0.020
Melero et al. (36)	0.26 (0.03, 0.49)	0.030	76%	<0.001
Pinto-Escalona et al. (38)	0.22 (−0.06, 0.50)	0.120	76%	<0.001

### 3.3 The effect of interventions on reading comprehension achievement

**Figure 5:**
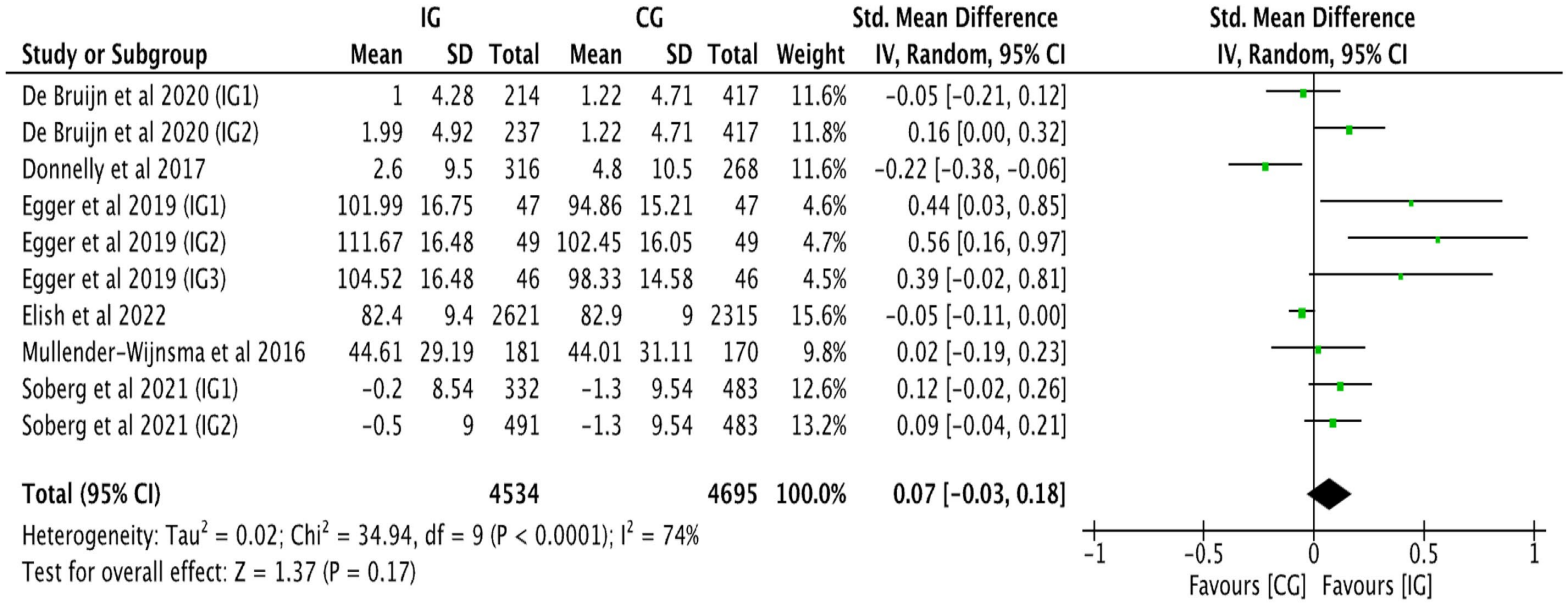
Forest plot of reading comprehension achievement.

**Figure 6:**
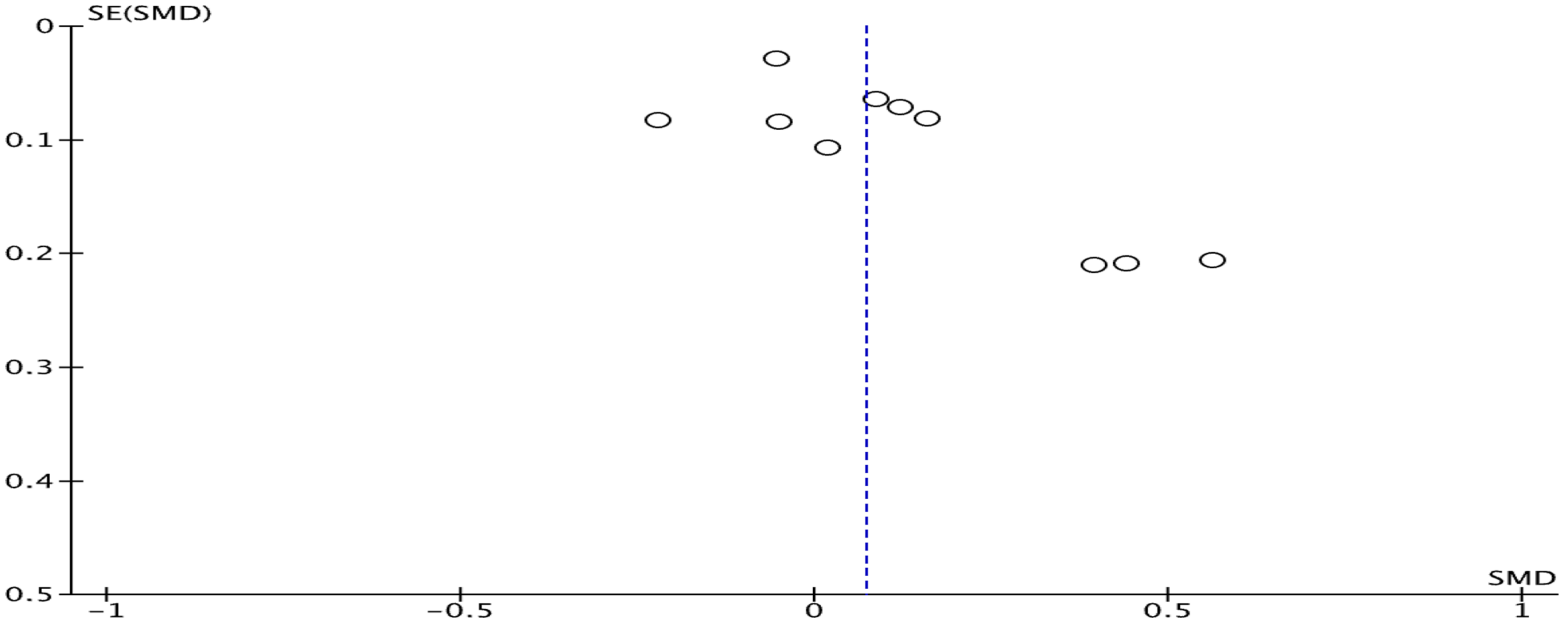
Funnel plot of reading comprehension achievement.

Figure 5 shows the combined effect size of six studies (27–30, 37, 40), indicating no significant correlation between physical activity and reading comprehension achievement (SMD = 0.07, 95% CI: −0.03 to 0.18, *p* = 0.170, *I^2^* = 74%). A subgroup analysis based on intervention duration was conducted (Table 2), indicating a positive association between physical activity and reading comprehension achievement when the intervention duration <24 weeks (SMD = 0.24, 95% CI: 0.03–0.46, *p* = 0.010; *I^2^* = 69%). A subgroup analysis based on intervention intensity was undertaken (Table 2), indicating a favorable connection between high intensity physical exercise and reading comprehension achievement (SMD = 0.50, 95% CI: 0.22–0.79, *p* < 0.001; *I^2^* = 0%). Sensitivity analysis found that heterogeneity remained considerable even after individual studies were eliminated (Table 3). Funnel plot analysis was undertaken to assess publication bias (Figure 6). The funnel plot for reading comprehension achievement showed broadly symmetrical, with no notable evidence of publication bias.

### 3.4 The effect of interventions on language expression achievement

**Figure 7:**
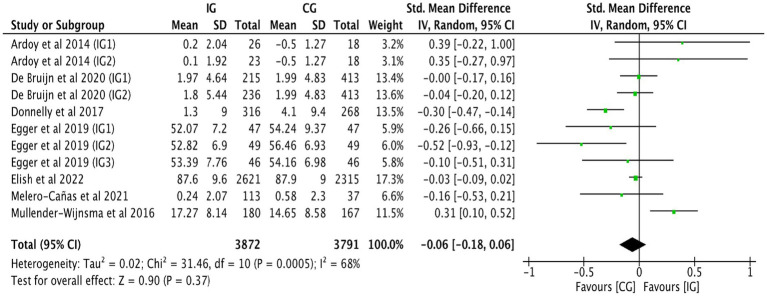
Forest plot of language expression achievement.

**Figure 8:**
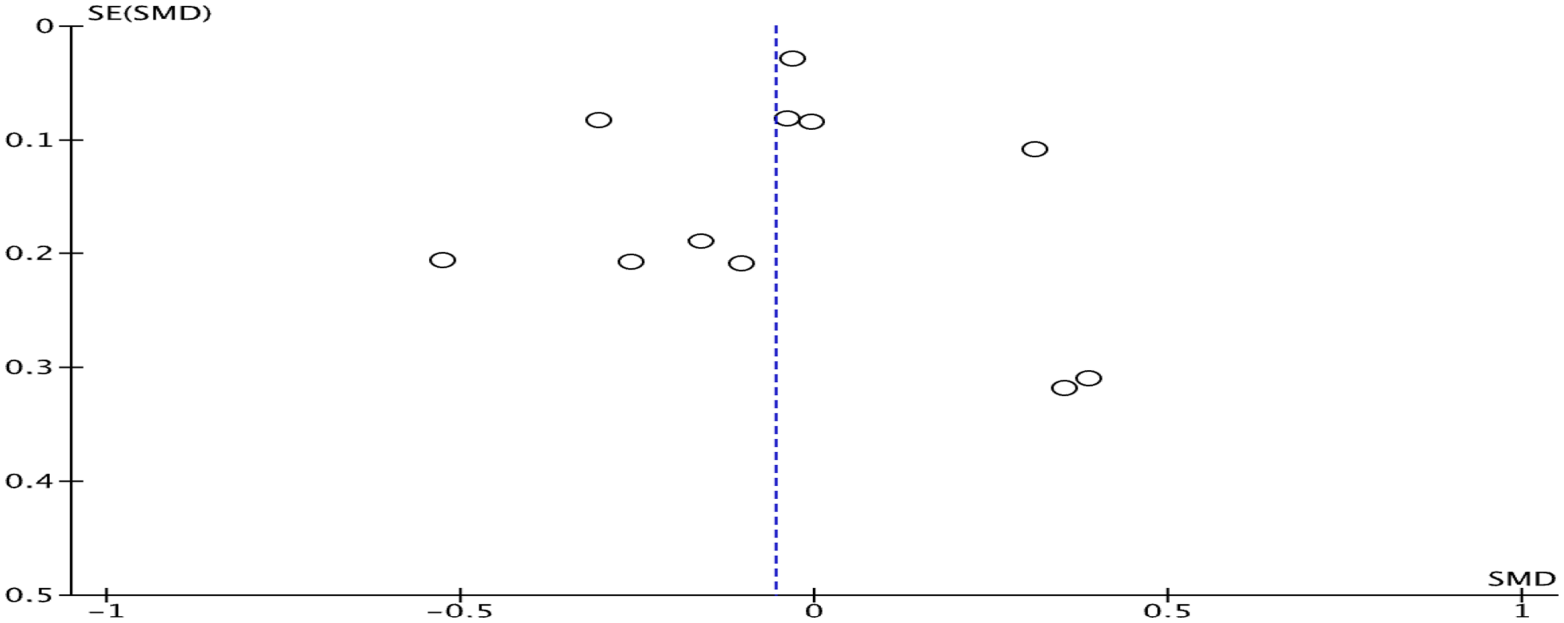
Funnel plot of language expression achievement.

Figure 7 shows the combined effect size of seven studies (25, 27–30, 36, 37), indicating no correlation between physical activity and language expression achievement (SMD = −0.06, 95% CI: −0.18 to 0.06, *p* = 0.370, *I^2^* = 68%). Heterogeneity remained high even after individual studies were excluded. A subgroup analysis regarding intervention length was performed (Table 2), revealing no link between physical activity and language expression achievement (SMD = −0.06, 95% CI: −0.18 to 0.06, *p* < 0.001; *I^2^* = 69%). A subgroup analysis regarding intervention intensity was performed (Table 2), revealing no link between physical activity and language expression achievement (SMD = −0.06, 95% CI: −0.18 to 0.06, *p* < 0.001; *I^2^* = 68%). Sensitivity analysis indicated that the exclusion of Donnelly et al. (28) diminished heterogeneity in the aggregated data (*I^2^* = 57%, *p* = 0.010). Excluding Mullender-Wijnsma et al. (37) also diminished heterogeneity (*I^2^* = 55%, *p* = 0.020), despite neither study displaying any distinctive characteristics. The heterogeneity in the other trials exhibited no significant alterations before and after elimination, as indicated in Table 3, and did not exert a discernible impact on the outcomes. Funnel plot analysis was employed to evaluate publication bias (Figure 8). The funnel plot for language expression achievement exhibited a nearly symmetrical shape, indicating no discernible publishing bias.

### 3.5 The effect of interventions on overall academic achievement

**Figure 9:**
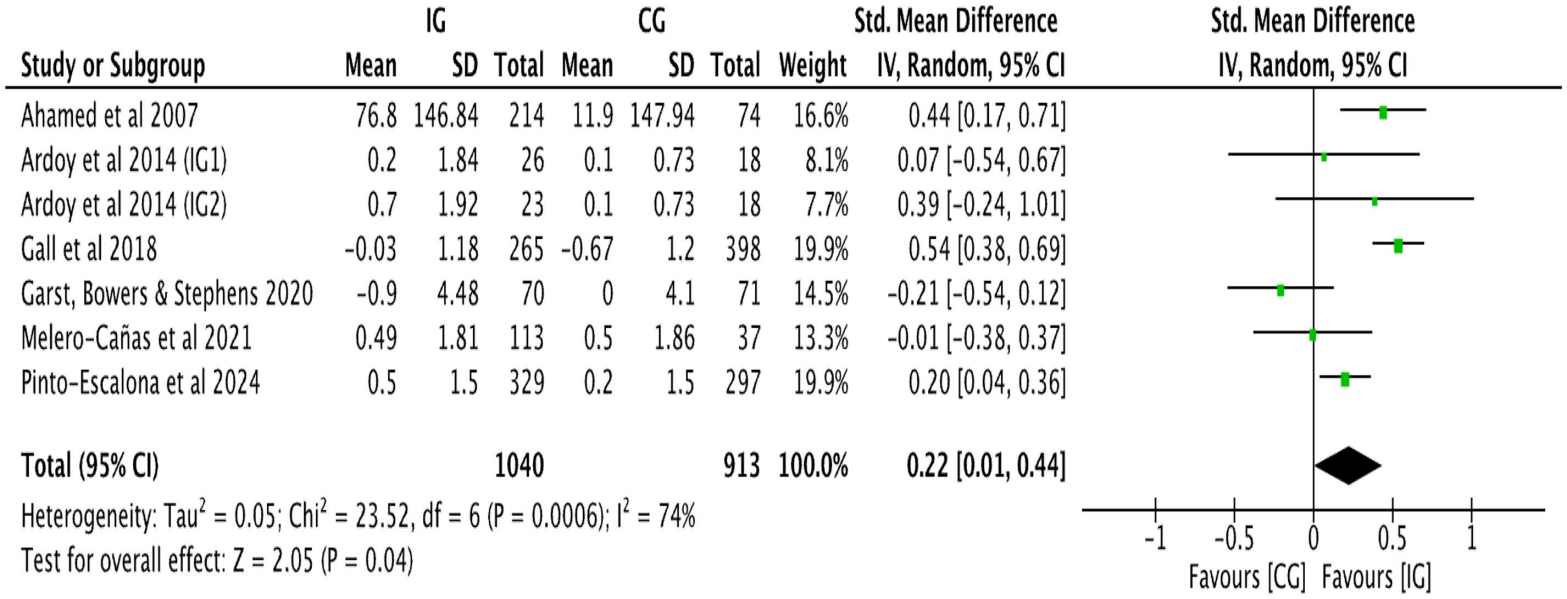
Forest plot of overall academic achievement.

**Figure 10:**
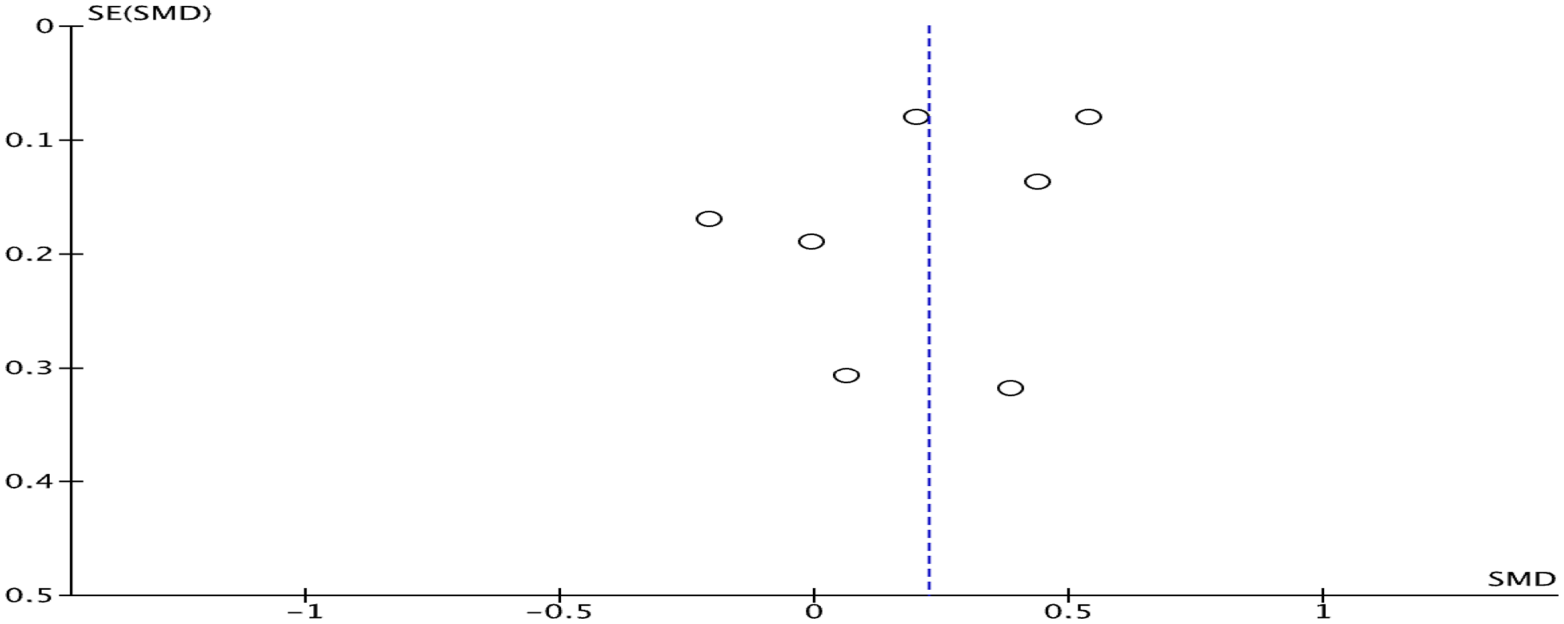
Funnel plot of overall academic achievement.

Figure 9 shows that the combined effect size of six studies (24, 25, 31, 32, 36, 41) was SMD = 0.22, 95% CI: 0.01–0.44, *p* = 0.040, indicating a significant positive impact of physical activity on overall academic achievement. There was considerable variability in effect sizes among the studies (*I^2^* = 74%, *p* < 0.001). A subgroup analysis was performed based on the duration of the intervention (Table 2). When the intervention duration was <24 weeks, no association was observed between physical activity interventions and overall academic achievement (SMD = 0.13, 95% CI: −0.11 to 0.37, *p* = 0.300; *I^2^* = 70%). However, when the intervention duration was ≥24 weeks, a positive association was found between physical activity and overall academic achievement (SMD = 0.47, 95% CI: 0.25–0.68, *p* < 0.001; *I^2^* = 14%). A subgroup analysis was performed based on the intervention’s intensity (Table 2). Moderate intensity physical activity has shown a favorable connection with overall academic achievement (SMD = 0.36, 95% CI: 0.14–0.44, *p* < 0.001; *I^2^* = 70%). High intensity physical exercise has shown a favorable connection with overall academic achievement (SMD = 0.22, 95% CI: 0.01–0.68, *p* < 0.001; *I^2^* = 74%). Sensitivity analysis revealed that the exclusion of Gall et al. (31) diminished heterogeneity in the aggregated results (*I^2^* = 52%, *p* = 0.060). This study did not demonstrate any distinctive traits. The heterogeneity in the other studies exhibited no significant alterations before and after elimination, as seen in Table 3, and did not substantially affect the results. This indicates that the findings of studies regarding the effects of physical activity interventions on overall academic achievement exhibit low sensitivity, signifying that the meta-analysis results are robust. Funnel plot analysis was employed to evaluate publication bias (Figure 10). The limited number of research on overall academic achievement hindered the assessment of the funnel plot’s symmetry and precluded the removal of publication bias.

## 4 Discussion

This review systematically evaluates the effectiveness of school-based physical activity interventions on academic achievement in children and adolescents. A total of 17 studies were included, and the overall results suggest that school-based physical activity interventions can improve academic achievement in children and adolescents, particularly in mathematics and overall academic performance. However, the evidence for improvements in reading comprehension and language expression remains inconclusive.

This study found a positive association between school-based physical activity and mathematics achievement. Integrating school-based physical activity with mathematics instruction has been shown to enhance students’ mathematics performance (35). School-based physical activity may improve mathematics achievement by increasing learning interest and reducing anxiety among students with high motor skills, while personalized tasks and differentiated instruction for students with lower motor skills can help optimize cognitive load (42). Recent meta-analyses have indicated that mathematics instruction incorporating physical activity can significantly improve mathematics achievement and simultaneously promote the development of students’ cognition, emotions, and motivation (43). Physical activity conducted during school hours has also demonstrated significant positive effects on overall academic achievement, with notable improvements in standardized test scores (44). Various forms of school-based physical activity, including cross-curricular physical activity, appear to have a stronger facilitating effect on academic performance (43). However, increasing school-based physical activity has not been shown to significantly improve standardized test scores in reading comprehension and language expression (45), which is consistent with our findings.

Although several academic outcomes demonstrated statistically significant improvements following school-based physical activity, the corresponding effect sizes were generally modest. This may reflect the multifactorial nature of academic performance, in which physical activity is only one of many contributing factors. Additionally, substantial heterogeneity was observed in the analyses of reading comprehension and overall academic achievement (*I*^2^ = 74% for both), indicating notable variability across studies. This heterogeneity may stem from differences in intervention duration, intensity, participant baseline academic levels, implementation fidelity, and assessment methods. While subgroup analyses revealed some favorable effects—particularly under short-term and high-intensity interventions—the limited number of included studies, especially the six focusing on overall academic achievement, warrants cautious interpretation of the pooled estimates. These limitations suggest that individual study characteristics may have disproportionately influenced the overall results. Therefore, statistical significance should be interpreted in the context of both effect magnitude and study-level variability. Our meta-analysis identified a small but statistically significant effect of school-based physical activity on overall academic achievement (SMD = 0.22, 95% CI: 0.01–0.44). This finding is consistent with a recent meta-analysis by Xu et al. (46), which reported a comparable effect size (SMD = 0.17, 95% CI: 0.02–0.32, *p* = 0.02) based on 13 studies examining classroom-based physical activity (CBPA). However, important differences exist between the two analyses. While Xu et al. focused exclusively on CBPA interventions, our review encompassed a broader range of school-based physical activity programs, including structured physical education and recess-based interventions. This wider scope may account for the higher heterogeneity observed in our findings. Moreover, our subgroup analyses yielded more granular insights, demonstrating that interventions lasting ≥24 weeks and involving moderate-to-high intensity were associated with larger and more consistent improvements in academic outcomes—an aspect not addressed in previous meta-analyses. These comparisons highlight the critical role of intervention characteristics in determining academic impact and underscore the added value of our study. Future research should aim to increase the number of high-quality studies, standardize intervention protocols and outcome measures, and employ moderator analyses to better identify the conditions under which school-based physical activity is most effective in enhancing academic outcomes.

In school physical education and activity practices, exercise load is considered one of the core concepts in kinesiology, with its key elements primarily involving exercise duration and intensity. As an important indicator for evaluating the effectiveness of school-based physical activity, exercise load plays a vital role in regulating instructional content, achieving educational goals, and ensuring students’ physical health. The results of subgroup analysis revealed a positive correlation between moderate-intensity physical activity and mathematics achievement, as well as overall academic performance. This is consistent with the findings of Berger and McInman (47), which demonstrated that moderate-intensity physical activity (20–60 min per session) contributes to emotional improvement (47). Research also indicates that incorporating moderate-to-high intensity physical activity into academic curricula, without reducing class time, increases the overall physical activity time in schools and enhances students’ academic achievement, while also establishing an innovative classroom model that integrates movement and learning (29).

This study found that increasing the intensity of physical activity in the school environment can have a positive impact on improving mathematics and overall academic performance. This may be because the intensity of school-based physical activity and students’ motivation or perceived engagement in class (i.e., behavioral, emotional, and cognitive participation) may serve as potential mediators of academic achievement in children and adolescents (48). However, school-based physical activity interventions were found to be most effective in enhancing mathematics achievement, particularly moderate-to-vigorous physical activity sustained for more than 6 months, which showed a significant overall effect size on mathematics scores. These findings indicate that certain intervention characteristics—particularly moderate-to-vigorous intensity and extended duration—may be more effective in enhancing academic performance. However, given the limited number of studies and substantial heterogeneity, these observations should be interpreted with caution. The small number of studies within each subgroup and the presence of substantial heterogeneity significantly limit the reliability and generalizability of these results. The observed trends may reflect preliminary associations rather than consistent, replicable effects. As such, current evidence on the dose–response relationship between school-based physical activity and academic achievement remains tentative. Future research should prioritize well-powered randomized controlled trials with standardized intervention protocols to more accurately determine threshold effects and identify optimal parameters for intervention design.

## 5 Limitation

While this study employed the *I^2^* statistic to quantify heterogeneity, the absence of meta-regression and sensitivity analyses—primarily due to the limited number of included studies and substantial variability in study characteristics—represents a notable methodological limitation. Here’s a refined version of the paragraph to make it more academic and reduce similarity: The heterogeneity observed in the studies can be attributed to several factors, including the variability of the intervention programs, differences in participant characteristics, and the use of diverse measurement tools. Moreover, regional cultural contexts and urban–rural disparities may serve as underlying and significant sources of this heterogeneity. The studies included in this review span diverse regions, such as North America, Europe, and Australia, where substantial differences in educational policies, sports culture, and the prioritization of the relationship between academic achievement and physical activity exist. Additionally, disparities in socio-economic conditions, as well as the significant variation in school sports infrastructure, teacher expertise, family support, and students’ extracurricular time between urban and rural areas, likely contribute to the inconsistent effects of the interventions observed across these studies. High levels of heterogeneity, particularly evident in outcomes related to reading comprehension and overall academic performance, compromise the precision and generalizability of the pooled estimates, and ultimately weaken confidence in the robustness of the conclusions. Moreover, several subgroup analyses were based on only two or three studies, substantially limiting statistical power and increasing the likelihood of unstable or spurious effect sizes. These constraints underscore the need for caution in interpreting subgroup findings. To enhance the reliability and interpretability of future meta-analytic work, there is a critical need for more rigorously designed studies with harmonized intervention protocols, standardized outcome assessments, and more homogeneous samples. Such improvements would facilitate the use of moderator analyses, including meta-regression, to identify systematic sources of between-study variability.

## 6 Conclusion

This study confirms the beneficial effects of school-based physical activity on academic achievement, particularly in improving mathematics achievement and overall academic achievement. Interventions lasting more than 6 months and performed at moderate to high intensity demonstrated the most significant impact on mathematics scores. Therefore, relevant authorities, educational institutions, and instructors are encouraged to implement school-based physical activity programs of at least 6 months in duration and moderate intensity to effectively enhance students’ academic achievement. However, no conclusive evidence was found regarding the impact on reading comprehension and language expression, which may depend on the type and duration of physical activity. Future research should further investigate these factors, as well as explore potential mediating mechanisms underlying the relationship between physical activity and academic achievement. In addition, a lack of standardized intervention protocols, limited long-term follow-up, and insufficient analysis of differential responses by age, gender, and baseline academic level remain key challenges. Lastly, the practical feasibility of implementing high-intensity interventions in typical school settings is limited by time and resource constraints, underscoring the need to develop more pragmatic and scalable models.

## Data Availability

The original contributions presented in the study are included in the article/supplementary material, further inquiries can be directed to the corresponding author.
